# Conditional Deletion of the *Pten* Gene in the Mouse Prostate Induces Prostatic Intraepithelial Neoplasms at Early Ages but a Slow Progression to Prostate Tumors

**DOI:** 10.1371/journal.pone.0053476

**Published:** 2013-01-08

**Authors:** Mi Kyung Kwak, Daniel T. Johnson, Chunfang Zhu, Suk Hyung Lee, Ding-Wei Ye, Richard Luong, Zijie Sun

**Affiliations:** 1 Department of Urology, Stanford University School of Medicine, Stanford, California, United States of America; 2 Department of Genetics, Stanford University School of Medicine, Stanford, California, United States of America; 3 Department of Comparative Medicine, Stanford University School of Medicine, Stanford, California, United States of America; 4 Department of Urology, Fudan University, Shanghai Cancer Center, Shanghai, People’s Republic of China; Florida International University, United States of America

## Abstract

The *PTEN* tumor suppressor gene is frequently inactivated in human prostate cancer. Using *Osr1* (*odd skipped related 1*)-*Cre* mice, we generated a novel conditional *Pten* knockout mouse strain, *Pten^LoxP^:Osr1-Cre*. Conditional biallelic and monoallelic *Pten* knockout mice were viable. Deletion of *Pten* expression was detected in the prostate of *Pten^LoxP/LoxP^:Osr1-Cre* mice as early as 2 weeks of age. Intriguingly, *Pten^LoxP/LoxP^*:*Osr1-Cre* mice develop high-grade prostatic intraepithelial neoplasms (PINs) with high penetrance as early as one-month of age, and locally invasive prostatic tumors after 12-months of age. *Pten^LoxP/+^*:*Osr1-Cre* mice show only mild oncogenic changes after 8-weeks of age. Castration of *Pten^LoxP/LoxP^*:*Osr1-Cre* mice shows no significant regression of prostate tumors, although a shift of androgen receptor (AR) staining from the nuclei to cytoplasm is observed in *Pten* null tumor cells of castrated mice. Enhanced Akt activity is observed in *Pten* null tumor cells of castrated *Pten^LoxP/LoxP^:Osr1-Cre*. This study provides a novel mouse model that can be used to investigate a primary role of *Pten* in initiating oncogenic transformation in the prostate and to examine other genetic and epigenetic changes that are required for tumor progression in the mouse prostate.

## Introduction

Prostate cancer affects about 2,276,000 men in the United States and is the most common non-cutaneous malignancy among males in the Western world (http://seer.cancer.gov/statfacts/html/prost.html). The phosphatidylinositol (3,4,5)-phosphate-kinase (PI3K) signaling pathway plays a critical role in human tumorigenesis, including prostate cancer [1]. The phosphatase and tensin homolog chromosome 10 (PTEN) is a tumor suppressor [2], [3] and functions as a negative regulator of the PI3K pathway by blocking the activation of the kinase Akt/PKB [4], [5]. PTEN is one of the most commonly mutated and/or deleted tumor suppressors in human malignancies. Somatic mutations of PTEN have been detected at high frequency in many sporadic cancers, including glioblastoma, endometrial cancer, and prostate cancer [6]. Complete PTEN inactivation has been found in 15% of primary prostate tumors, and in up to 60% of prostate cancer metastases [7], [8], [9].

The biological significance of *Pten* has been carefully characterized in numerous mouse models. *Pten* null (*Pten* ^−/−^) mice are embryonic lethal [10]. *Pten* heterozygous (*Pten* ^+/−^) mice are viable and develop tumors in a variety of organs, including the breast, thyroid, endometrium, and prostate [11], [12], [13]. Biallelic conditional knockout of *Pten* in mice induces tumor development in specific tissues [14]. Mouse models with conditional *Pten* deletion in the prostate have been developed in the past decade [15], [16], [17]. In particular, deletion of *Pten* in the mouse prostate using probasin-*Cre* induces oncogenic transformation [15]. In mice, loss of one allele of *Pten* is associated with the development of high-grade prostatic intraepithelial neoplasia (PIN) and the loss of both alleles of *Pten* results in invasive prostate cancer that metastasizes to lymph nodes and the lung [15]. *Pten* null tumors in *Pten^LoxP/LoxP^:PB-Cre4* mice can further develop to androgen independent cancer after castration [15].

An interaction between the androgen and PI3K/AKT signaling pathways has been implicated in prostate tumorigenesis [18], [19]. Recent studies have shown that loss of *Pten* expression represses androgen action in *Pten* knockout mice [20], [21]. Elimination of murine prostatic *Pten* expression in these mouse models is mediated through *Cre* activation driven by the probasin promoter, in which *Cre* expression is postnatal and androgen-dependent [22]. In this study, we developed a novel conditional *Pten* knockout mouse strain using newly developed *Osr1* (*odd skipped related*) *Cre* mice [23]. *Osr1*-*Cre* activity is detected in mouse urogenital organs, including the prostate, bladder, and kidney at E11.5, although it also becomes active in the endoderm of the posterior foregut, midgut, and hindgut. Intriguingly, *Pten^LoxP/LoxP^*:*Osr1-Cre* mice develop high-grade prostatic intraepithelial neoplasms (PINs) as early as 4-weeks of age and prostatic tumors after 12-months of age. *Pten ^LoxP/+^*:*Osr1-Cre* mice show only mild oncogenic changes in the prostate. Castration of *Pten^LoxP/LoxP^*:*Osr1-Cre* mice shows no significant regression of prostate tumors, although a shift of AR staining from the nuclei to cytoplasm is observed. This study describes an innovative and additional mouse model for assessing a primary role of *Pten* in initiating oncogenic transformation as well as identifying additional genetic and epigenetic changes that are required for promoting tumor progression in the mouse prostate.

## Materials and Methods

### Generation and Genotyping of the Conditional *Pten* Knockout Mice

Mice homozygous for floxed *Pten* exon 5, *Pten^loxP/loxP^,* on a 129/Balb/c background, were obtained from the Jackson Laboratory (Strain#: 004597, Bar Harbor, ME). They were crossed with the *Osr1-Cre* strain in an *FVB/N* background, which was a kind gift of Dr. Gail Martin at UCSF [23], and *PB-Cre4* mice on a *C57BL/6xDBA2* background, in which the *Cre* transgene is controlled by a modified probasin promoter (ARR2PB) [22]. F1 *Pten^LoxP/+^:Osr1-Cre* mice were backcrossed more than five times with C57BL/6J mice and then used for mating to produce both heterozygous (*Pten^LoxP/+^:Osr1-Cre*) and homozygous (*Pten^loxP/loxP^:Osr1-Cre*) mice. Using similar mating strategies, we also generated *Pten* conditional knockout mice with *PB-Cre4* mice. Littermate controls lacking the *Cre* transgene were used in all experiments. All animal experiments performed in this study were approved by the ethics committee of the Administrative Panel on Laboratory Animal Care at Stanford University.

Mice were genotyped by PCR as described previously [15]. Mouse tail-tips were isolated and incubated overnight at 55°C in lysis buffer (Cat# 102-T, VIAGEN Biotech, LA, CA*)* with 0.5 µg/mL proteinase K (Cat# 03-H5-801-001, Roche Diagnostics, Indianapolis, IN). Tail-tip samples were then incubated at 85°C for 45 min before use. The forward primer (5′-TCCCAGAGTTCATACCAGGA-3′) and the reverse primer (5′-AATCTGTGCATGAAGGGAAC -3′) were used to distinguish the wild type and target alleles by amplifying the flanking *loxP* sites. The forward primer (5′-TTGCCTGCATTACCGGTCGATGCA-3′) and the reverse primer (5′-GATCCTGGCAATTTCGGCTAT-3′) were used to detect the *Cre* transgene. Genomic DNA fragments were amplified at 95°C for 5 min, then 95°C for 45 sec, 58°C for 40 sec, and 72°C for 60 sec for 36 cycles, then 72°C for 5 min. For detection of exon 5 deletion, genomic DNA samples were isolated from different mouse organs using similar methods as for mouse tail-tips. The forward primer (5′-ACTCAAGGCAGGGATGAGC-3′), and reverse primer, (5-GCTTGATATCGAATTCCTGCAGC-3′) were used to detect exon 5 deletion as reported previously [15].

### Histological Analyses and Immunohistochemistry

Mouse tissues were fixed in 10% neutral-buffered formalin and processed into paraffin for standard histology and immunohistochemistry. Samples were cut into 5 µm thick sections, deparaffinized in xylene, and rehydrated using a decreasing ethanol gradient followed by 0.1 M phosphate buffer saline (PBS). Tissues were then blocked with 0.3% hydrogen peroxide in methanol for 15 min. Tissues were then blocked with 5% goat serum in PBS for 30 minutes at room temperature (RT). Tissue sections were exposed to a 1∶100 dilution of anti-mouse/human PTEN antibody (Cascade Bioscience, Inc., 6H2.1), 1∶500 dilution of anti-mouse AR (Santa Cruz, sc-816), 1∶250 dilution of anti-p63 antibody (Santa Cruz, sc-8431), 1∶3000 of anti Ki67 antibody (Novacastra, NCL-ki67), 1∶300 of E-cadherin antibody (Transduction Laboratories, c20820), 1∶800 of CK-5 antibody (Covance, PRB-160P), 1∶800 of CK8 antibody (Covance, MMS-162P), 1∶200 of synaptophysin antibody (Invitrogen, 18-0130), 1∶50 pAkT antibody (Cell Signaling Technologies, 4060), or 1∶50 AKT (Cell Signaling Technologies, 9272) in 1% goat serum at 4°C overnight. Slides were then incubated with biotinylated anti-rabbit or anti-mouse secondary antibody (Vector Laboratories, BA-1000 or BA-9200) for 1 h, horseradish peroxidase streptavidin (Vector Laboratories, SA-5004) for 30 min at room temperature, and then visualized by DAB kit (Vector Laboratories, SK-4100). Slides were subsequently counterstained with 5% (w/v) Harris Hematoxylin.

### Western Blotting

Mouse tissues were isolated from different organs and homogenized in ice-cold RIPA buffer (150 mM sodium chloride, 1% NP-40, 0.5% sodium deoxycholate, 0.1% SDS, 50 mM Tris, pH 8.0, 1 mM PMSF, 1 µg/ml aprotinin, 1 µg/ml leupeptin). Protein concentrations were measured using a protein assay kit (Bio-Rad Laboratories, Cat#: 500-0006). Protein samples were resolved through 8% SDS-polyacrylamide gel and transferred to nitrocellulose membrane. After blocking in 5% nonfat dry milk, membranes were probed with primary antibody specific for PTEN at 1∶500 (Cell Signaling). A 1∶2000 dilution of ß-actin antibody was used to normalize protein samples. Protein detection was performed with ECL reagents according to the manufacturer’s protocol using ECL Hyperfilm (Amersham Biosciences).

### Statistical Analyses

We present the data as the mean±SD. We made comparisons between groups, using a two-sided Student’s *t* test. P<0.05, P<0.01, P<0.001 were considered significant.

## Results

### Conditional Deletion of *Pten* in Mouse Prostate with *Osr1-Cre*

**Figure 1:**
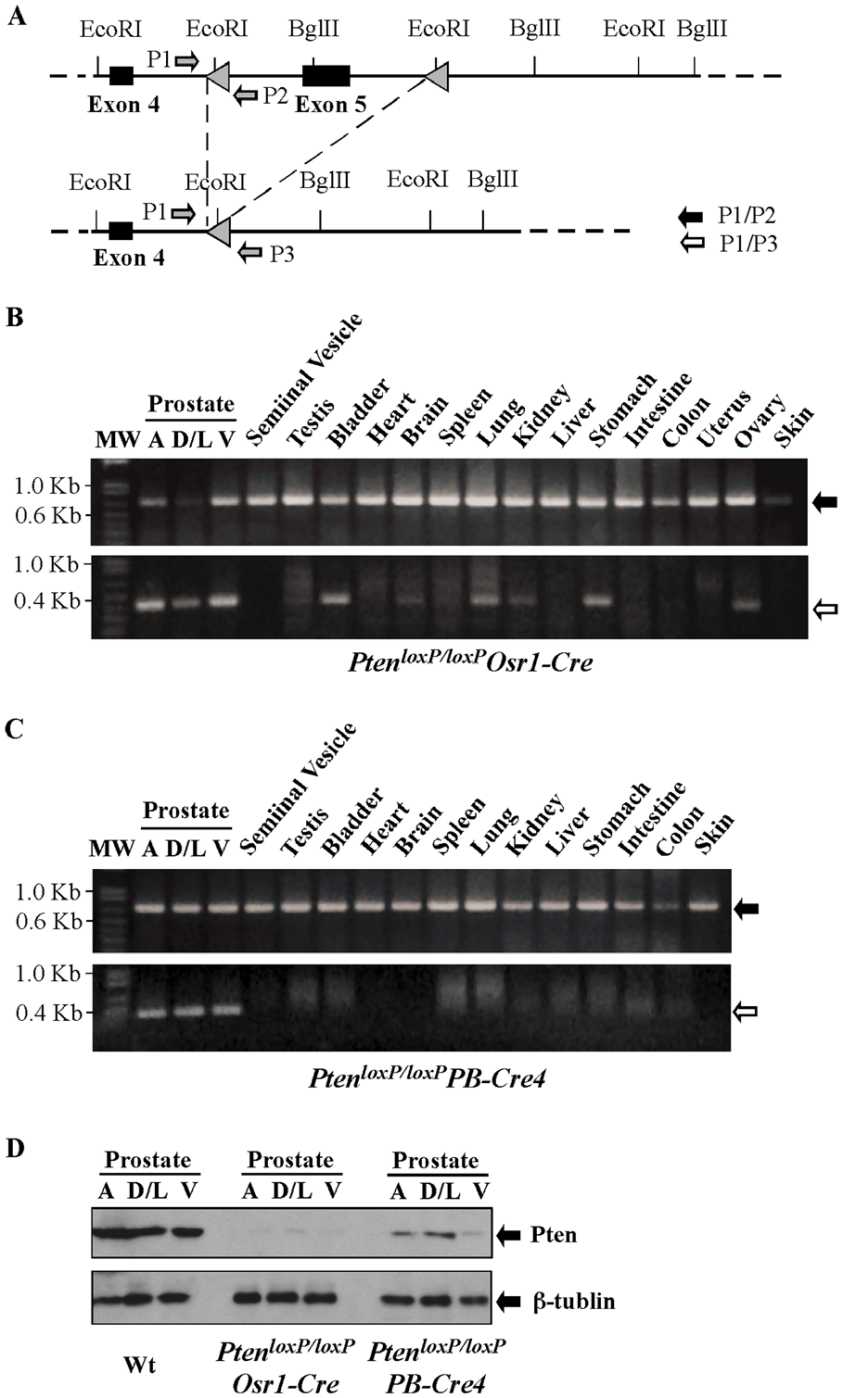
Generating and analyzing *Pten Osr1-Cre* conditional knockout mice. **A.** Diagram for *Pten* knockout strategy. In *Pten^loxP/loxP^* mice, *LoxP* recognition sequences were inserted into the endogenous *Pten* locus flanking exon 5 as previously reported [15]. Two different Cre transgenic lines carrying *Osr1-Cre* or *PB-Cre4* transgene were crossed to *Pten^loxP/loxP^* mice for the generation of *Pten^loxP/loxP^:Osr1-Cre* and *Pten^loxP/loxP^;PB-*Cre4 mice. **B and C.** Genomic PCR analyses for *Pten^loxP/loxP^*:*Osr1-Cre* and *Pten^loxP/loxP^*:*PB-Cre4* mice. PCR was performed using genomic DNA samples harvested from various organs in *Pten^loxP/loxP^*:*Osr1-Cre* and *Pten^loxP/loxP^*:*PB-Cre4* mice. Primers in the *Pten* locus were used to detect either the *loxP* allele or the *Cre*-recombined allele. The 0.65 kb fragment of the *Pten loxP* allele (solid arrows) was observed in all 15 organs from both *Pten^loxP/loxP^*:*Osr1-Cre* and *Pten^loxP/loxP^*:*PB-Cre4* mice. The 0.3kb Cre-recombined fragment (open arrows) was detected only in the prostate of *Pten^loxP/loxP^:PB-Cre4* mice and in the prostate, urinary bladder, brain, lung, kidney, stomach, and ovary of *Pten^loxP/loxP^*:*Osr1-Cre* mice. **D.** Western blotting to detect *Pten* expression in *Pten^loxP/loxP^*:*Osr1-Cre* and *Pten^loxP/loxP^*:*PB-Cre4* mice. Whole cell lysates isolated from prostate lobes were analyzed by Western blot with the PTEN antibody. b-actin was used as a loading control. AP, anterior; DLP, dorsolateral and VP, ventral prostate lobes.

The *Osr1* promoter activates at E11.5 in urogenital sinus epithelium and retains its activity in prostatic epithelium throughout development [23]. Because mouse endogenous Pten is expressed prenatally during the course of prostate development [24], we used a newly established *Osr1* (*odd-skipped-related 1*) *Cre* line to examine the effect of *Pten* conditional deletion during the course of prostate development. We made the conditional *Pten* knockout mice by crossing *Pten* floxed mice with *Osr1-Cre* mice (Fig. 1A). To test the efficiency of *Osr1-Cre* mediated *Pten* deletion in different mouse tissues, we performed genomic PCR analyses using DNA samples isolated from various mouse tissues from 2-week old male and female *Pten^loxP/loxP^:Osr1-Cre* mice. The specific deletion of *Pten* exon *5* by *Osr1-Cre* was observed in all prostatic lobes in *Pten^loxP/loxP^:Osr1-Cre* mice (Fig. 1B). *Cre*-mediated *Pten* deletion was also detected in other organs and tissues of *Pten^loxP/loxP^:Osr1-Cre* mice, including the urinary bladder, lung, stomach, ovary, testis, brain, spleen, and kidney. However, there was no detectable *Cre*-mediated activity in other organs, such as the seminal vesicle, heart, liver, intestine, colon, uterus, and tail. These results indicate that *Osr1-Cre* activity is not limited to the mouse prostate gland, which is consistent with previous reports [23]. In a parallel control experiment, we observed a specific effect of *PB-Cre4* mediated *Pten* deletion in age- and sex-matched *Pten^loxp/loxp^:PB-Cre4* control mice (Fig. 1C). However, *Cre*-mediated *Pten* deletion appears more efficient in the prostate gland of *Pten^loxP/loxP^:Osr1-Cre* mice than *Pten^loxP/loxP^:PB-Cre4* mice (Fig. 1B and 1C). Using Western-blot, we further assessed expression of *Pten* in the prostate gland of 4-week old *Pten^loxP/loxP^:Osr1-Cre* mice. A marked reduction of PTEN expression appeared in four different prostatic lobes of *Pten^loxP/loxP^:Osr1-Cre* mice (Fig. 1D). Decreased PTEN expression was also observed in all prostatic lobes of *Pten^loxP/loxP^:PB-Cre4* mice, however the reduction in ventral lobes appeared more pronounced (Fig. 1D).

### *Osr1-Cre* Mediated *Pten* Deletion Leads to Oncogenic Transformation of the Mouse Prostate

**Figure 2:**
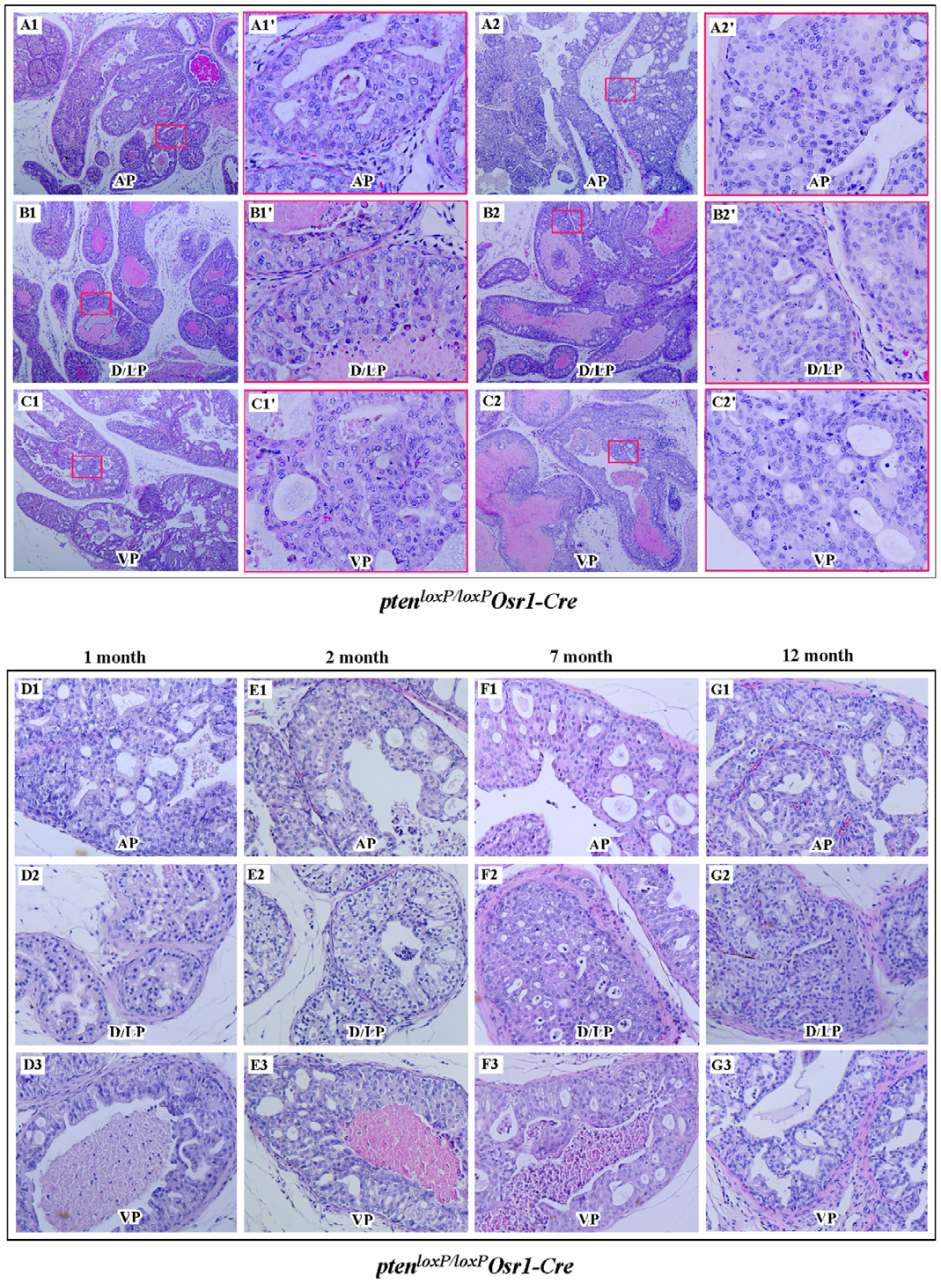
Development of PIN lesion in *Pten^loxP/loxP^:Osr1-Cre* mice. **A–C.** H&E staining for 2 to 4 week old *Pten^loxP/loxP^ Osr1-Cre* mice. Representative images of mouse PIN lesions are shown from different prostate lobes of 2 week old *Pten^loxP/loxP^*:*Osr1-Cre* mice; AP, anterior; D/LP, dorso/lateral, and VP, ventral prostate lobes. Immunohistochemical analyses of these samples show the PIN lesions with concurrent absence of *Pten* expression (please see Figure S1). The typical high-grade PIN lesions are mainly cribriform or stratified in nature, and often distended the prostate glandular unit profile and/or completely filled the prostate glandular unit lumen. Mitoses were rare. The fibromuscular stroma remained intact, with overall glandular unit profiles being recognizable. **D–G.** Histological analyses of the prostate tissues isolated from different age groups of *Pten^loxP/loxP^*:*Osr1-Cre* mice. However, the nature and extent of the high grad PIN in *Pten^loxP/loxP^:Osr1-Cre* mice did not appear to change with advancing age, as evidence by the similarities of lesions in the 4-week old mice as compared to their 2-, 7-, and 12-month of cohorts.

Both *Pten^LoxP/+^:Osr1-Cre* and *Pten^loxP/loxP^:Osr1-Cre* mice were born at the expected Mendelian ratios, suggesting that there is no significant prenatal lethality associated with these mice. All *Pten* conditional knockout mice appear normal and do not show significant differences in appearance with age-matched *Pten^LoxP/+^*and *Pten^loxP/loxP^* littermates, or wildtype controls. We monitored the survival rate of both male and female *Pten^LoxP/+^:Osr1-Cre* and *Pten^loxP/loxP^:Osr1-Cre* mice as well as controls for 12 months, and did not see a significant difference between the groups (data not shown). In an effort to search for phenotypes of these *Pten* knockout mice, we examined the conditional knockout mice at 1-, 2-, 4-, 7-, 12-, and after 12-months of age. Histologically, we observed proliferative lesions consistent with prostatic intraepithelial neoplasia (PIN) in both *Pten^LoxP/+^:Osr1-Cre* and *Pten^loxP/loxP^:Osr1-Cre* mice. Strikingly, we detected high-grade PIN lesions in *Pten^loxP/loxP^:Osr1-Cre* mice as early as one-month of age (Table 1). We observed 4 of 4 (100%) *Pten^loxP/loxP^:Osr1-Cre* mice to have developed high-grade PIN lesions in all glandular units of each prostatic lobe, including the anterior prostate gland (Fig. 2A1and 2A2), the dorsal/lateral prostate gland (Fig. 2B1 and 2B2), and ventral prostate gland (Fig. 2C1 and 2C2). These high-grade PIN lesions appeared as atypical epithelial cells forming cribriform structures (Fig. 2A1′; 2C1′, 2A2′, 2B2′, and 2C2′) and/or with stratification (Fig. 2B1′), and often distended the prostate glandular unit profile and/or completely filled the prostate glandular unit lumen. The atypical cells displayed mild anisocytosis and mild pleocytosis, with abnormal nuclear polarity and nuclear crowding. Mitoses, however, were rarely observed. Additionally, in each mouse, the fibromuscular stroma was intact, with overall glandular unit profiles remaining recognizable (Fig. 2A1, 2B1, and 2C2), albeit enlarged and distended. We observed very similar high-grade PIN lesions in the prostates of *Pten^loxP/loxP^:Osr1-Cre* mice at different ages (Fig. 2D to 2G). There was no significant pathological change, in terms of disease progression, in the four prostatic lobes between different age groups of *Pten^loxP/loxP^:Osr1-Cre* mice. Using an antibody against Pten, we confirmed the loss of *Pten* expression within these PIN lesions in *Pten^loxP/loxP^:Osr1-Cre* mice (Figure S1). These results provide a direct link between the loss of *Pten* protein expression and the development of dysplastic lesions within the prostate gland of *Pten^loxP/loxP^:Osr1-Cre mice*. In the *Pten^loxP/loxP^:PB-Cre4* mouse model, it has been shown that high grade PIN lesions progress to invasive prostatic cancer within 4 to 5 months. However, we did not observe invasive tumors in the prostate glands of *Pten^loxP/loxP^:Osr1-Cre* mice younger than 12-months old (Table. 1). We also did not observe any notable gross or pathological changes in prostate tissues isolated from newborns of *Pten^LoxP/LoxP^:Osr1-Cre* and *Pten^LoxP/+^:Osr1-Cre mice* (data not shown). In addition, we examined non-prostatic pathogenic abnormalities in both male and female *Pten^LoxP/+^:Osr1-Cre* and *Pten^loxP/loxP^:Osr1-Cre* mice. As detailed in Table S1, we observed malignancies and other abnormalities in approximately 10% of *Pten^loxP/loxP^:Osr1-Cre* mice older than 10 months old. However, this study specifically focuses on the pathological changes of the prostate glands of *Pten^loxP/loxP^:Osr1-Cre* mice.

**Table 1:** Pathological Abnormalities in the Prostates of Targeted *Pten* Knockout mice.

Genotypes	1 month	2 to 4 months	7 to 9 minths	>12 months
*Controls*	5 of 5 normal	9 of 9 normal	10 of 10 normal	8 of 8 normal
*Pten ^loxP/+^:Osr1-Cre*	1 of 4 hyperplasia	2 of 7 focal PIN	6 of 8 focal PIN	7 of 8 focal PIN
			3 of 8 high grade PIN	5 of 8 high grade PIN
*Pten ^loxP/loxP^:Osr1-Cre*	4 of 4 high grade PIN	7 of 7 high grade PIN	8 of 8 high grade PIN	8 of 8 high grade PIN
				5 of 8 carcinomas

### Conditional Deletion of the *Pten* Gene in Mouse Prostatic Cells Activates the AKT Signaling Pathway and Induces Prostatic Cell Proliferation

**Figure 3:**
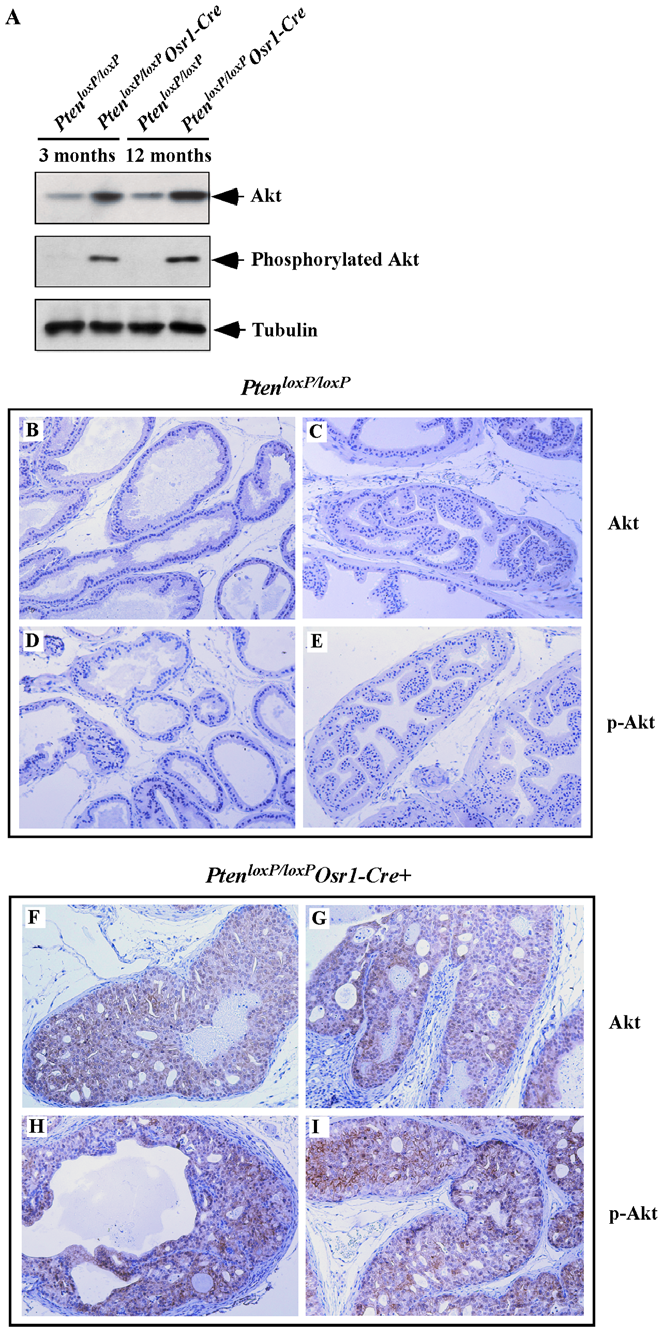
Deletion of *Pten* increases expression and activity of Akt in *Pten^loxp/loxp^:Osr1-Cre* mice. **A.** Analysis of Akt and pAkt expression in prostate tissues of 3 and 12-month old *Pten^loxP/loxP^:Osr1-Cre* and *Pten^loxP/loxP^* mice. Westernblotting was performed to examine both Akt and phospho-Akt expression. β-tubulin was used as a loading control. **B–I.** Immunohistochemical analysis of Akt and phospho-Akt in prostate sections. Akt expression levels are increased in the epithelial cells within high-grade PIN regions of *Pten^loxP/loxP^*:*Osr1-Cre* prostates (**F, G**), compared with *Pten^loxP/loxP^* mice (**B, C**). Activation of Akt was assessed with a phospho-Akt antibody. High levels of phospho-Akt immunoreactivity was observed in the high grade PIN regions of *Pten^loxP/loxP^*:*Osr1-Cre* prostates (**H, I**) compared with the low signal observed in the prostate epithelial cells of *Pten^loxP/loxP^*:prostates (**D, E**).

It has been shown that inactivation of *Pten* function induces the activation of the PI3K/AKT signaling pathway in human cancer cells [25], [26]. Previous prostate *Pten* conditional knockout mouse models displayed increased PI3K/AKT signaling [15]. To address the role of *Pten* on regulating Akt signaling, we surveyed expression and activity of AKT, a major downstream target of the PI3K signaling pathway, in prostate tissues isolated from *Pten^loxP/loxP^:Osr1-Cre* mice. Increased staining of Akt was observed in prostate tissues isolated from both 3- and 12-month old *Pten^loxP/loxP^:Osr1-Cre* mice in comparison with those from age matched *Pten^loxP/loxP^* control mice (Fig. 3A). Blotting with the phosphorylated-Akt antibody also showed elevated activation of Akt signaling in the prostate tissues of *Pten^loxP/loxP^:Osr1-Cre* mice (Fig. 3A). We further assessed expression and activity of Akt in the prostate tissues of both *Pten^loxP/loxP^:Osr1-Cre* and *Pten^loxP/loxP^* mice using immunohistochemistry. We observed strong immunoreactivity of Akt in high-grade PIN lesions in *Pten^loxP/loxP^:Osr1-Cre* mice (Fig. 3F and 3G) but either no or low expression of Akt in the prostate tissues of *Pten^loxP/loxP^* control mice (Fig. 3B and 3C). Using a phosphorylated-Akt antibody, we further assessed the activation of Akt in the above tissues. Immunoreactivity of phosphorylated Akt appeared in *Pten^loxP/loxP^:Osr1-Cre* mice (Fig. 3H and 3I) but not in *Pten^loxP/loxP^* control mice (Fig. 3D and 3E). This data demonstrates the activation of the Akt signaling pathway in *Pten* null cells of *Pten^loxP/loxP^:Osr1-Cre* mice.

**Figure 4:**
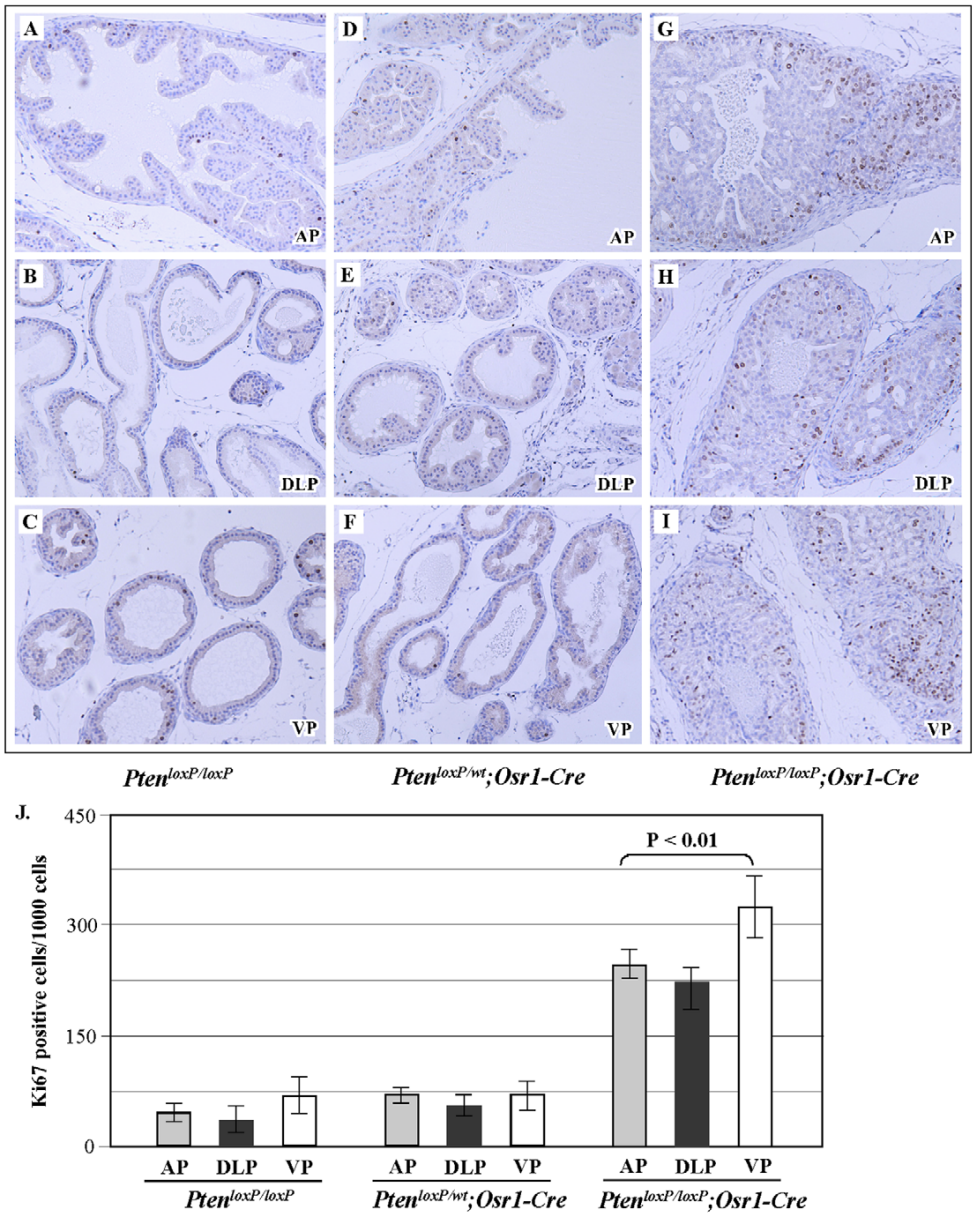
Deletion of *Pten* induces cell proliferative advantage in prostate tissues. **A–I.** Cell proliferation was examined by immunostaining for Ki-67. Prostate sections isolated from 4-week-old *Pten^loxP/loxP^*, *Pten^loxP/+^:Osr1-Cre,* and *Pten^loxP/loxP^ Osr1-Cre* mice were stained for Ki-67. A total of 1000 epithelial cells in each lesion from three different lesions from three mice of each genotype were evaluated for Ki-67 immunoreactivity. **J.** The positive immunoreactive cells for Ki-67 in *Pten^loxP/loxP^*:*Osr1-Cre+* mice are significantly greater than both *Pten^loxP/loxP^* control mice and *Pten^loxP/wt^:Osr1-Cre* mice, P<0.01.

A promotional role of the PI3K/AKT pathway in cell survival and proliferation has been implicated, which is negatively regulated by PTEN [1], [15]. To assess the effects resulting from conditional knockout of *Pten* in the mouse prostate on cellular proliferation in prostate tissues, we used Ki67 immunohistochemistry on prostate tissues from *Pten^loxP/loxP^:Osr1-Cre*, *Pten^loxP/+:^Osr1-Cre*, and *Pten^loxP/loxP^* mice. Specifically, a cellular proliferation index was quantified by evaluating a total of 1000 epithelial cells for Ki67 nuclear immunoreactivity in three different areas/lesions each from 3 different mice of each genotype. A significant increase in cellular proliferation was observed in high-grade PIN lesions of *Pten^loxP/loxP^:Osr1-Cre* mice in comparison to normal prostate tissues of *Pten^LoxP/+^:Osr1-Cre* and *Pten^loxP/loxP^* mice (Fig. 4A to 4I). The cellular proliferation index increased from approximately 100 in cells in *Pten^loxP/loxP^* and *Pten^loxP/+^:Osr1-Cre* mice to more than 200 in PIN lesions of *Pten^loxP/loxP^:Osr1-Cre* (P<0.01, Fig. 4J) mice. These results indicate an inhibitory role of *Pten* in prostatic cell proliferation.

### Analysis of Cellular Markers of Atypical Cells within PIN Lesions

**Figure 5:**
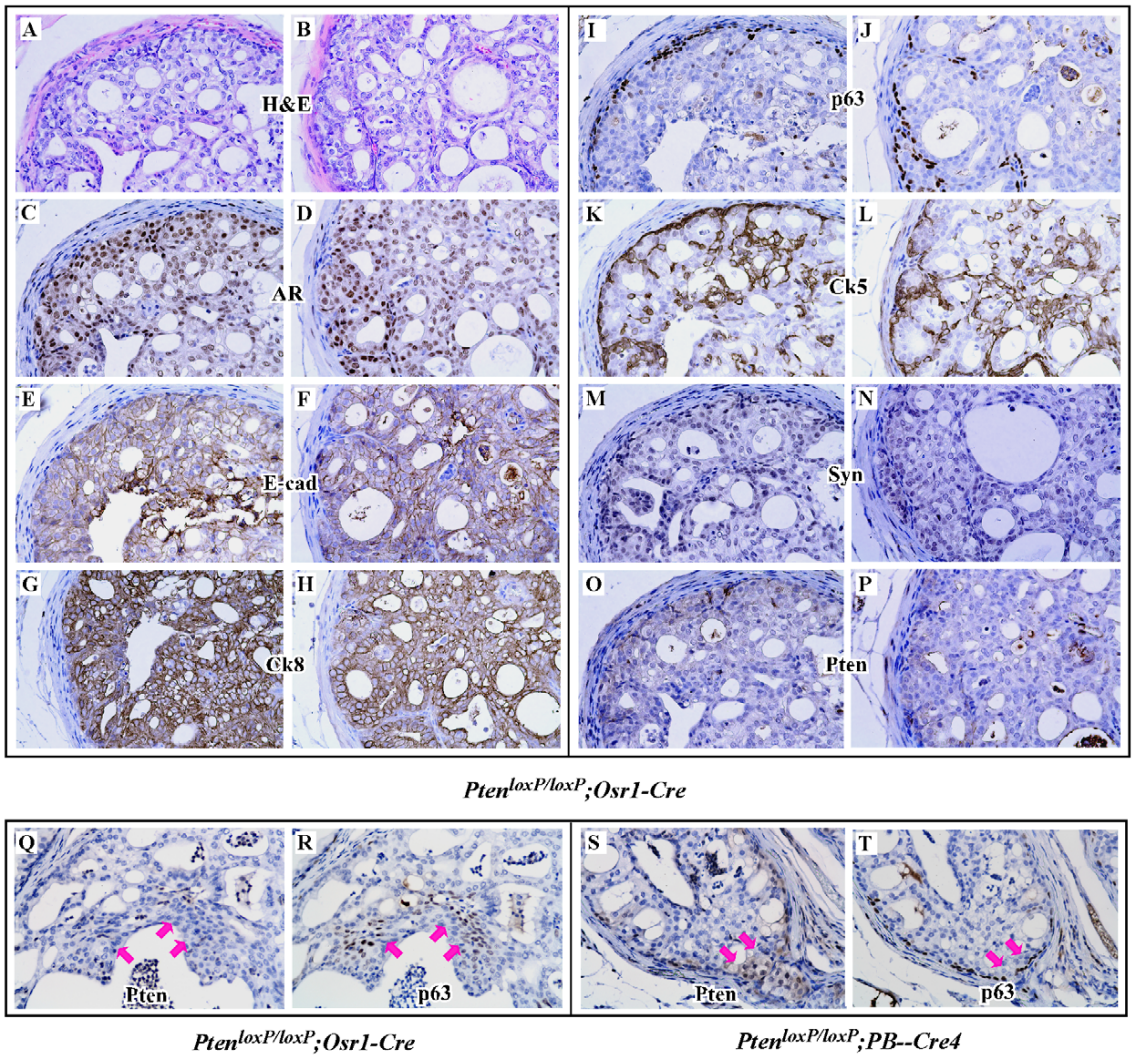
Immunohistochemistry analyses of the prostate tissues isolated from *Pten^loxP/loxP^:Osr1-Cre*. H&E staining and immunohistochemistry were performed on prostate tissues of 4-week-old *Pten^loxP/loxP^*:*Osr1-Cre* mice. Adjacent prostate tissue sections from two *Pten^loxP/loxP^*:*Osr1-Cre* mice were analyzed with different antibodies as marked, including the AR (**C, D**), CK8 (**G, H**), E-cadherin (**E, F**), synaptophysin (**M,N**), p63 (**I, J**) and CK5 (**K, L**). A pair of adjacent slides from the prostate tissues of either *Pten^loxP/loxP^:Osr1-Cre* or *Pten^loxP/loxP^:PB-Cre4* mice were subjected to immunohistochemistry using the Pten and p63 antibodies. Representative images focusing on p63 positive cells (arrows) are shown (**Q–T**).

Mouse prostatic epithelium is composed of several cell types, including basal and luminal epithelial cells, as well as neuroendocrine cells. Development of prostatic adenocarcinoma in mouse prostates has been demonstrated in previous *Pten* prostate conditional knockout mice generated using *ARR2PB-Cre* mice [15]. Activity of the *Osr1* promoter has been detected at E11.5 in the urogenital sinus and prostatic epithelium of developed prostate glands [23]. To further define the origin of tumors in these *Pten* knockout mice, we performed comprehensive immunohistochemical analyses to examine a series of prostatic cellular markers on these high-grade PIN lesions (Fig. 5). Atypical cells of PIN lesions failed to immunoreact with Pten antibody (Fig. 5O to 5P), suggesting a direct link between depletion of *Pten* expression and oncogenic transformation in the prostate of the knockout mice. Most atypical prostatic cells showed typical nuclear immunoreactivity with AR (Fig. 5C and 5D). Atypical cells also showed positive immunoreactivity for E-cadherin and CK8 (Fig. 5E to 5H), secretory epithelial markers, but showed no immunoreactivity for the neuroendocrine cell marker synaptophysin (Fig. 5 M and 5N). Immunoreactivity for p63, a cellular marker for prostatic basal epithelial cells, appeared mainly in the basal compartment of normal prostatic glands, but rarely in atypical cells (Fig. 5I and 5J). Interestingly, positive immunoreactivity of CK5 was also observed in some atypical cells within PIN lesions (Fig. 5K and 5L). To confirm Pten deletion in prostate epithelium, we further stained adjacent slides prepared from *Pten^loxP/loxP^:Osr1-Cre* mice with both p63 and Pten antibodies. We observed no immunoreactivity to the Pten antibody in p63 positive cells in tissue slides prepared from *Pten^loxP/loxP^:Osr1-Cre* mice (Fig. 5Q and 5R). However, positive staining with the Pten antibody appears in p63 positive cells in samples prepared from age and sex matched *Pten^loxP/loxP^:PB-Cre4* controls. Using a double-fluorescent *mT/mG Cre reporter mouse strain*, we further examined *Osr1-Cre* activity in mouse prostatic epithelium [27]. Intriguingly, we observed clear cellular membrane staining of mG proteins in p63 positive cells of *Osr1-Cre* mice (Figure S2). Taken together, these data demonstrate that *Osr1-Cre* can be activated in basal epithelial cells of the mouse prostate.

### Development of Prostatic Adenocarcinoma in *Pten* Conditional Mice

**Figure 6:**
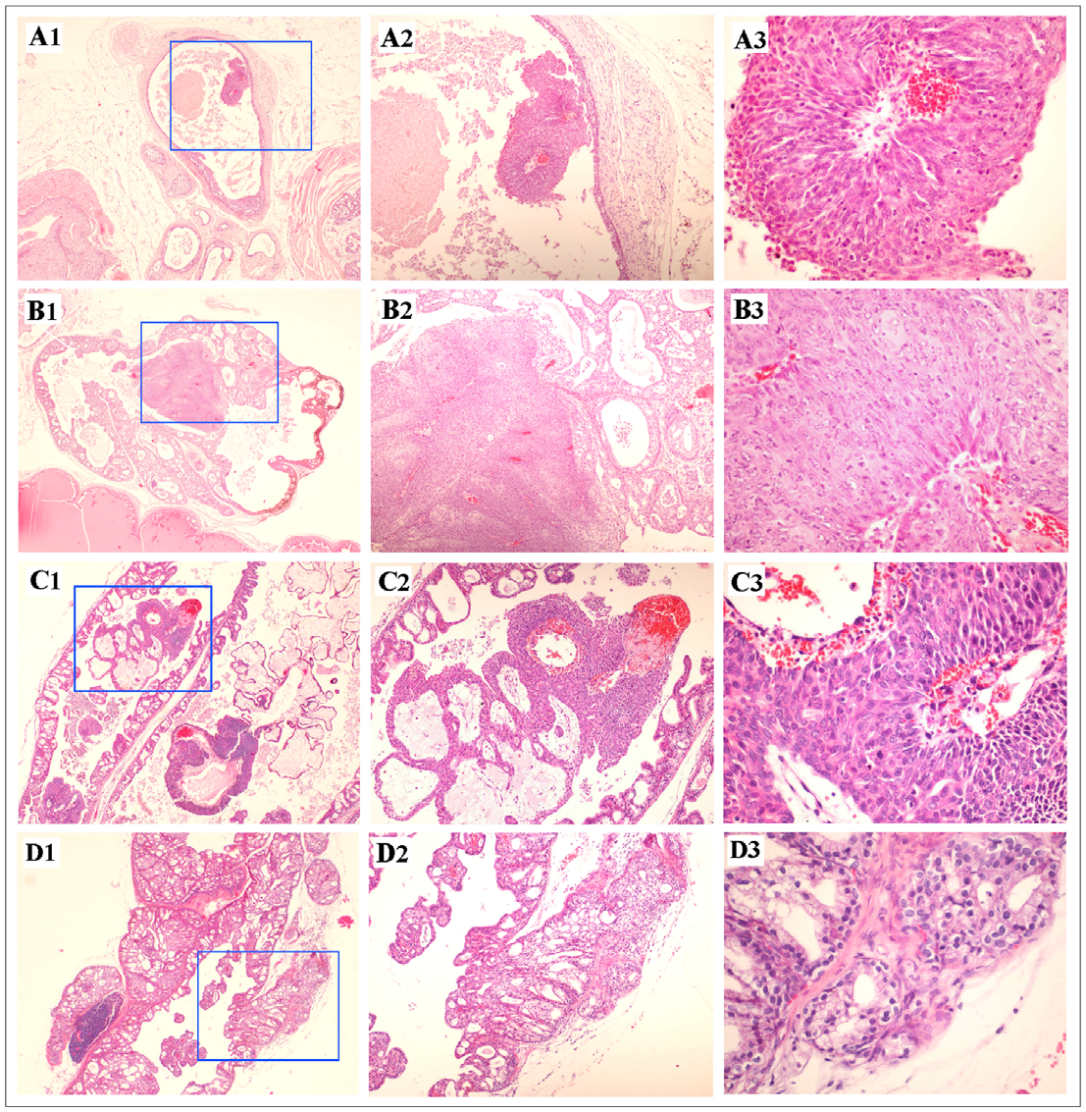
Development of malignant prostate tumors in older *Pten^loxP/loxP^:Osr1-Cre* mice. H&E staining was performed to analyze prostate tissues isolated from greater than 12-month-old *Pten^loxP/loxP^:Osr1-Cre* mice. Intra-glandular solid prostatic carcinomas were observed in 5 mice and three were shown here (**A to C**), each of which appear wholly contained within enlarged prostatic glandular units that otherwise contain abundant proliferative changes consistent with high grade PIN. In addition to increased cellular and nuclear atypia, an observable increase in mitotic activity is noted in the solid carcinoma areas. No evidence for invasion beyond the basement membrane or fibromuscular stroma was noted. In another single mouse, invasion of a cribriform high grade PIN lesion beyond the basement membrane and fibromuscular stroma of the glandular unit was observed (**D**), which meets the criteria for a diagnosis of well-differentiated prostatic adenocarcinoma.

There is consensus that high grade PINs can progress towards prostate adenocarcinomas [28]. A previous study has shown that *Pten^loxP/loxP^:PB-Cre4* mice developed prostatic invasive adenocarcinomas between 9 to 29 weeks of age [15]. Interestingly, we continued to observe high-grade PIN lesions in *Pten^loxP/loxP^:Osr1-Cre* mice in 12-month-old mice, which are similar in morphology and extent to those observed in their younger counterparts. In 5 of 8 of these older mice, we also observed limited malignant progression of focal areas of high grade PIN lesions to prostatic carcinomas or adenocarcinomas, which is significantly higher than *Pten^loxP/+^:Osr1-Cre* and wild type littermates (Fig. 6A1 to 6D3). Specifically, there was intra-glandular malignant transformation in 4 of these 5 mice (Fig. 6A1 to 6C3; one not shown), represented by the development (in each case) of solid carcinoma, characterized by haphazard sheets of cells with scant fibrovascular stroma. The cells displayed more pronounced anisocytosis and pleomorphism than the surrounding high grade PIN tissues, as well as marked anisokaryosis and clearly observable mitoses (ranging from 1 to 4 per 400×field). However, in each case, the solid carcinoma was entirely contained within a markedly enlarged prostate glandular unit, with no observable invasion beyond the basement membrane of the overall glandular unit. In the remaining mouse (1 of 5 mice), a focal area of cribriform high grade PIN was observed to infiltrate beyond the basement membrane and fibromuscular wall of the glandular unit, and into surrounding serosal tissue (Fig. 6D1 to 6D3). This meets the criteria for malignant transformation of high grade PIN to well-differentiated prostatic adenocarcinoma, despite the limited area involved.

### Effects of Castration on *Pten* Null Prostate Tumors

**Figure 7:**
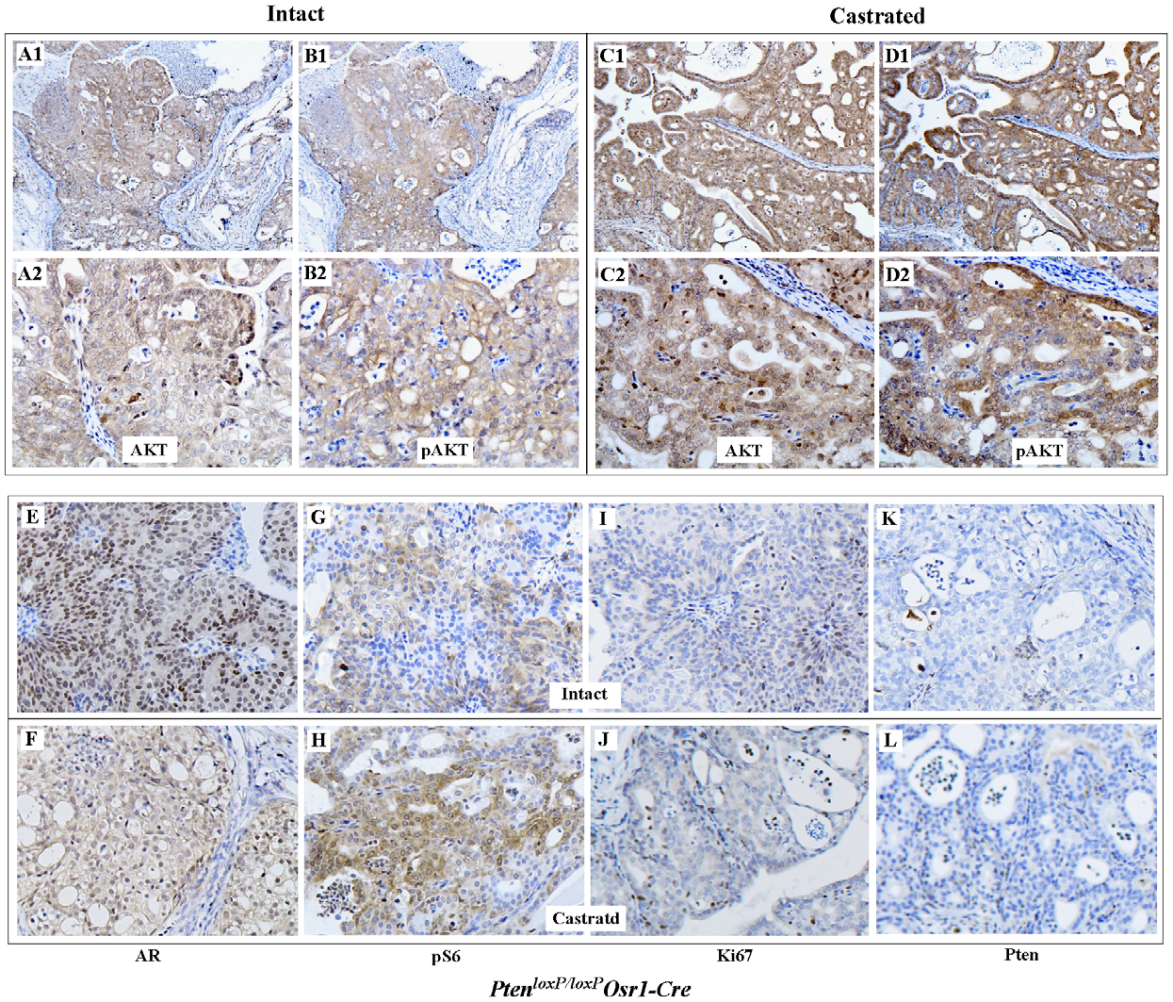
Examining AKT and AR signaling pathways in castrated *Pten-*null tumors. Immunohistochemical characterization was carried out to assess Akt and pAkt expression in *Pten* null prostate tumors isolated from intact (**A, B**) and castrated (**C, D**) 12-month-old *Pten^loxP/loxP^*:*Osr1-Cre* mice. The same tissue samples were also analyzed with the antibodies against, the AR, pS6, Ki67, and Pten immunohistochemcially (**E to L**).

Although early studies have shown that androgens are critical for the survival and proliferation of Pten null cancer cells in prostate knockout mouse models [15], a recent study has shown that castration has a very limited effect on inhibiting *Pten* null tumor formation and progression [21]. To assess the effect of androgen ablation on *Pten* null tumors of *Pten^loxP/loxP^:Osr1-Cre* mice, we castrated *Pten^loxP/loxP^:Osr1-Cre* mice at ages between 12 to 16 months. We carefully examined castrated mice at 10 weeks after castration with age matched controls, and found substantial PIN lesions in all four (4/4) castrated mice. There appears to be no difference in pathological changes between intact and castrated *Pten^loxP/loxP^:Osr1-Cre* mice (data not shown). Recent studies have shown that the AR and PI3K/AKT signaling pathways inversely regulate each other in *Pten* null tumor cells [20], [21]. Using specific antibodies against AKT and phosphorylated-AKT, we evaluated expression and activity of AKT in both intact and castrated *Pten^loxP/loxP^:Osr1-Cre* mice. While both AKT and pAKT staining appears in the lesions of both intact and castrated mice, intensive immunoreactivity to AKT and pAKT antibodies is observed in castrated mice (Fig. 7A1–B2 versus 7C1–D2). Staining of the above samples with an antibody against phosphorylated S6, a downstream target of AKT, also shows strong immunoreactivity in the lesions of castrated mice (Fig. 7G). A typical nuclear staining pattern of AR appears in tumor cells of intact mice (Fig. 7E), and in contrast a diffuse and cytoplasmic staining pattern of AR in castrated mice (Fig. 7F). There is no significant change in the staining of Ki67 between castrated and intact mice (Fig. 7I and 7J). These data are consistent with previous reports and suggests a negative role of androgen signaling in regulating the PI3K/AKT pathway in *Pten* null tumor cells [20], [21].

## Discussion

The PI3K/AKT pathway plays a critical role in prostate cell proliferation and survival [1]. PTEN, a tumor suppressor, is the most frequent altered protein in human prostate cancer [2], [4], [6], [10]. The major biological function of PTEN is to counteract PI3 kinase in regulating the phosphatidylinositol (3,4,5)-phosphate level in the cell. More evidence has shown that PI3K/AKT and PTEN affect cell growth and tumor formation *in vitro* and *in vivo* [4], [5]. In past years, significant effort has been put into characterizing the biological significance of PTEN in tumorigenesis using *Pten* knockout mouse models [29]. Several *Pten* conditional knockout mouse models have been established through inactivation of *Pten* by the *Cre-loxP* system. Particularly, *Pten^loxP/loxP^:PB-Cre4* mice, generated using a modified rat probasin promoter driven *Cre* expression, develop an interesting prostatic tumor phenotype, which has been frequently used in the field of prostate cancer research [15]. While loss of only one functional allele of *Pten* results in various stages of hyperplasia and dysplasia, no prostate cancer lesions are observed in these mice. It has been reported that loss of both alleles of *Pten* results in invasive prostate cancer as early as 9-weeks of age, often with metastases to lymph nodes and the lung [15]. Another prostate *Pten* conditional knockout mouse model was generated using human prostate specific antigen (PSA) promoter driven *Cre* expression [16]. Heterozygous deletions of *Pten* in the mice showed focal and low grade PIN at 10-months of age. Mice with homozygous deletions of *Pten* develop hyperplasia and focal PIN at 4- to 5-months of age, PIN with focal microinvasion at 7- to 9-months of age, and prostatic carcinomas at 10- to 14-months of age [16]. Only rare metastatic lesions were observed in this mouse model. These prostate *Pten* conditional knockout mice have shown different phenotypes in terms of the timing of disease development and progression as well as the occurrence of invasive or metastatic tumors.

In this study, we use a newly developed *Osr1-Cre* mouse strain to examine inactivation of *Pten* in the prostate gland. The *Osr1* promoter is active at E11.5 in urogenital sinus epithelium and maintains its activity in prostatic epithelial cells throughout development [23]. *Osr1-Cre* activity was detected in all four prostatic lobes in 2-week-old mice. As previously reported, *Osr1-Cre* activity was also detected in other mouse organs and tissues of *Pten^loxP/loxP^:Osr1-Cre* mice [23]. A remarkable reduction of Pten expression in all four prostatic lobes was detected using both Western-blotting and immunohistochemisty assays. As a consequence of inactivating *Pten* expression, we identified atypical proliferative lesions consistent with murine PIN in both *Pten ^LoxP/+^:Osr1-Cre* and *Pten^loxP/loxP^:Osr1-Cre* mice. We observed extensive and diffuse high grade PIN lesions as early as in one-month-old *Pten^loxP/loxP^:Osr1-Cre* mice. These pathological changes, however, appeared static in nature as the mice aged, appearing with similar morphology in mice up to 12-months of age. We only observed malignant prostatic tumors in *Pten^loxP/loxP^:Osr1-Cre* mice between 12–16 months of age. These findings suggest that the *Pten^loxP/loxP^:Osr1-Cre* strain is a new additional model that can be used to assess *Pten*-mediated oncogenic transformation in prostate tumorigenesis.

As previously reported, *Pten^loxP/loxP^:PB-Cre4* mice develop PIN after 6- to 8-weeks of age, and invasive tumors as early as 2-months of age [15]. In contrast, *PSA-Cre:Pten^loxP/loxP^* mice showed a slower tumor development phenotype: developing PIN at 4- to 5-months of age and prostatic adenocarcinoma at 10- to 14-months of age. Inactivation of *Pten* using MMTV-*Cre* transgenic mice showed completely penetrant high grade PIN development by 2-weeks of age, which frequently progressed to invasive adenocarcinomas by 7- to 14-weeks of age [17]. Based on these observations, it has been speculated that deletion of *Pten* in the prostate gland during or after puberty may increase the initiation, development, and progression of PIN towards malignant prostatic tumors. However, our *Pten^loxP/loxP^:Osr1-Cre* mice display an early onset of high grade PIN at 2-weeks of age, with more malignant prostatic tumors not developing until ages greater than 12-months, and with no signs of metastatic disease. These phenotypes suggest that inactivation of *Pten* at early ages may also be able to trigger oncogenic transformation and induce PIN lesions in the prostate with other additional “hits”, as observed in human prostate tumorigenesis [30]. As suggested by previous studies, these changes can be either *Pten*-dependent or -independent [17], [31], [32]. Although hyperplasia and PIN lesions have been frequently observed in *Pten* conditional knockout mouse models, the appearance of more malignant phenotypes varies greatly between the different models. Therefore, this new *Pten* knockout mouse model may help us to investigate the biological role of PTEN during the course of prostate cancer initiation and progression.

Using immunohistochemical approaches, we further analyzed tumor cells in PIN and prostatic carcinoma/adenocarcinoma lesions of *Pten^loxP/loxP^:Osr1-Cre* mice. We observed that most tumor cells in PIN and prostatic carcinoma/adenocarcinoma lesions expressed E-cadherin and were CK8 positive, but did not express synaptophysin. These data suggest that tumor cells are immunoreactive to luminal epithelial cellular markers. Interestingly, we also observed some immunolabeling with CK5 antibodies in PIN lesions in *Pten^loxP/loxP^:Osr1-Cre* mice. These data suggest that oncogenic transformation may be initiated in both basal and luminal epithelial cells through the inactivation of PTEN signaling. Using the double-fluorescent mT/mG Cre reporter mouse strain, we further confirmed that Osr1-*Cre* mediated recombination occurs in both luminal and basal epithelial cells of the mouse prostate (Figure S2). Our observations are consistent with previous studies showing that both prostatic luminal and basal epithelial cells are competent to function as tumor initiating cells [33], [34].

The promoting role of the PI3K and AKT pathways in inducing prostatic cell growth has been implicated in human prostate tumorigenesis. Inactivation of Pten in previous *Pten* knockout mouse models has shown increased cell proliferation. We also observed increased cellular proliferation based on increased Ki67 index in all PIN and prostatic prostatic carcinoma/adenocarcinoma samples. In this study, we also examined cell apoptosis in samples with PIN and prostatic carcinoma/adenocarcinoma lesions, but did not observe any significant changes. These data further support a repressive role of PTEN in cell proliferation.

Both *Pten^LoxP/+^:Osr1-Cre* and *Pten^loxP/loxP^:Osr1-Cre* mice were born at expected Mendelian ratios. We did not observe significant prenatal lethality associated with genotypes of these mice. While we observed some abnormalities in *Pten^loxP/loxP^:Osr1-Cre* mice at ages greater than 12-months (please see Table S1), it is unclear whether they are directly resulted from *Pten* deletion or due to other non-specific factors. *Osr-1* promoter activity appears to be active in other organs and tissues during development, which is consistent with previous reports [23]. Therefore, it is conceivable that deletion of *Pten* by *Osr1-Cre* may directly contribute to those changes. More detailed studies should be carried out to characterize the phenotypes of *Pten^loxP/loxP^:Osr1-Cre mice*.

The probasin promoter has been widely used to create prostate genetically-engineered mouse models. It is activated postnatally in an androgen-inducible manner [22]. Castration of conditionally-inactivated *Pten* mice using Probasin-Cre results in cancer regression, however tumors can become ligand-independent. In this study, we also castrated *Pten^loxP/loxP^:Osr1-Cre* mice at 12- to 16-months of age when prostatic carcinoma/adenocarcinoma develops. Ten weeks after castration, we found *Pten* null tumors to be pathologically similar to *Pten* null tumors in age-matched intact mice. Interestingly, castrated *Pten* null tumors showed a dominant cytoplasmic AR expression pattern, in contrast with the nuclear expression pattern observed in intact mice. We also observed strong AKT signaling in castrated *Pten* null mice, which is consistent with similar observations in *Pten^loxP/loxP^:PB-Cre4* mice [20], [21]. Deletion of *Pten* using *Osr1-Cre* provides another novel and useful model for investigating PTEN as a tumor suppressor in prostate cancer development and progression. Particularly, this new mouse model provides several unique features that will help us to further dissect the signaling pathways regulated by functional *Pten* loss during the course of prostate cancer initiation and progression.

## Supporting Information

Figure S1 Examining *Pten* expression. Prostate tissues isolated from 4-week-old *Pten^loxP/loxP^:Osr1-Cre* and *Pten^loxP/loxP^* control mice were subjected to histological and immunohistochemical analyses. Representative images from different prostatic lobes, including AP, anterior; D/LP, dorsolateral, and VP, ventral prostate lobes, are shown.
(TIF)

Figure S2 Generating mT/mG reporter mouse strains. **A.** Schematic diagram of the mT/mG reporter construct before and after Cre-mediated recombination. **B.** Live whole mount and fixed prostate tissues isolated from 8–12 week old male *mT/mG;Osr1-Cre* mice were analyzed. Representative Images were taken from prostate lobes, anterior (AP), dorsal (DP), lateral (LP), and ventral prostate (VP) of a 8 week old *mT/mG:Osr1-Cre* mice. **C.** Images were taken from a 8-week old make *mT/mG:PB-Cre* mice. **D.** Prostate tissues samples isolated from *mT/mG:Osr1-Cre* mice (**B**) were stained with the p63 antibody (red). Double p63 (red) and mG (green) positive cells are label with yellow arrows.
(TIF)

Table S1 Non-prostatic abnormalities in *Pten* conditional knockout mice.
(TIF)
