# Supplementary figures and images for: Conditional Deletion of the Pten Gene in the Mouse Prostate Induces Prostatic Intraepithelial Neoplasms at Early Ages but a Slow Progression to Prostate Tumors
Source: PLoS One. 2013 Jan 8;8(1):e53476. doi: 10.1371/journal.pone.0053476 (PMC3540073; doi:10.1371/journal.pone.0053476)

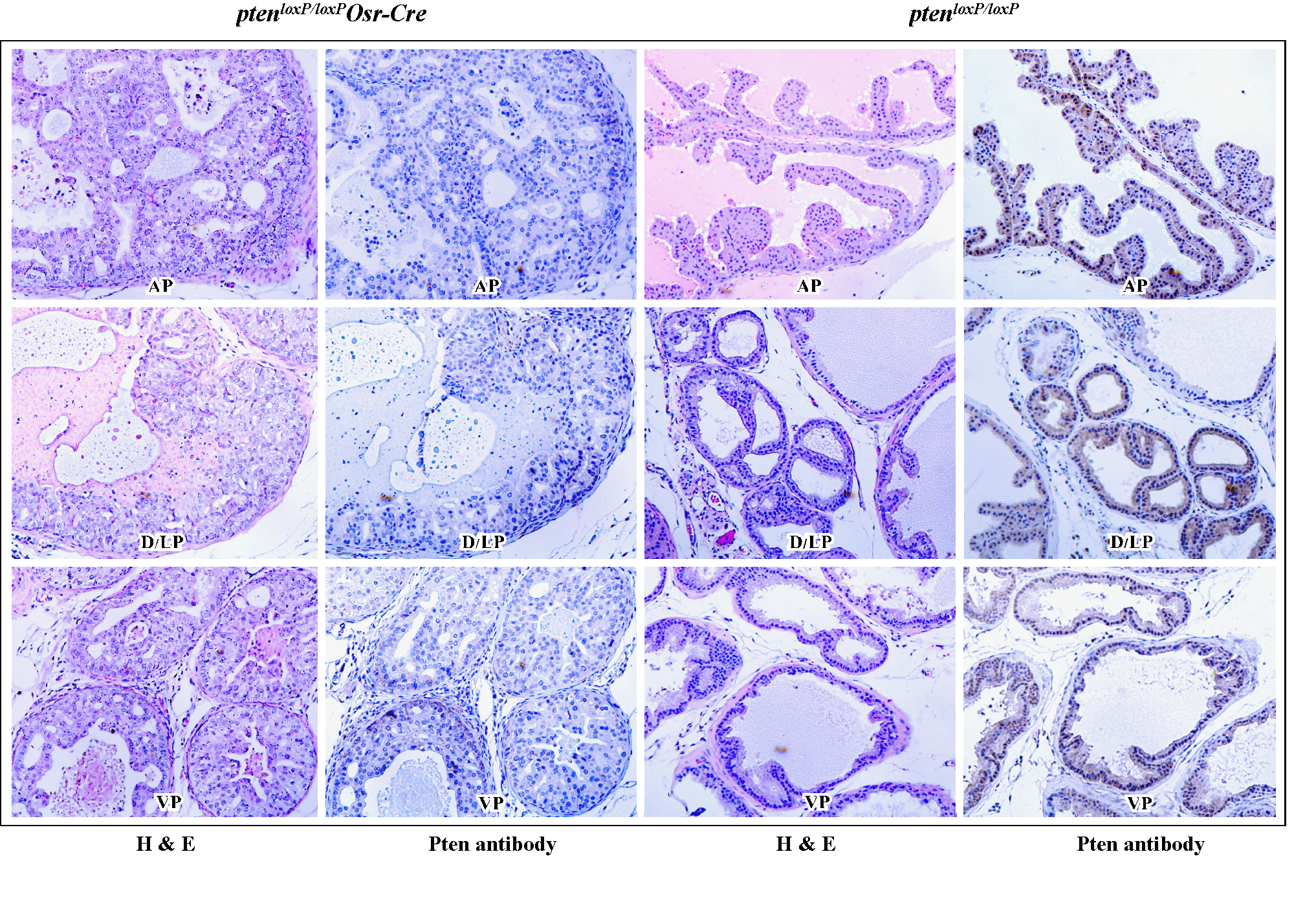

Supplement: Figure S1 — Examining Pten expression. Prostate tissues isolated from 4-week-old PtenloxP/loxP:Osr1-Cre and PtenloxP/loxP control mice were subjected to histological and immunohistochemical analyses. Representative images from different prostatic lobes, including AP, anterior; D/LP, dorsolateral, and VP, ventral prostate lobes, are shown. (TIF) [file pone.0053476.s001.tif]

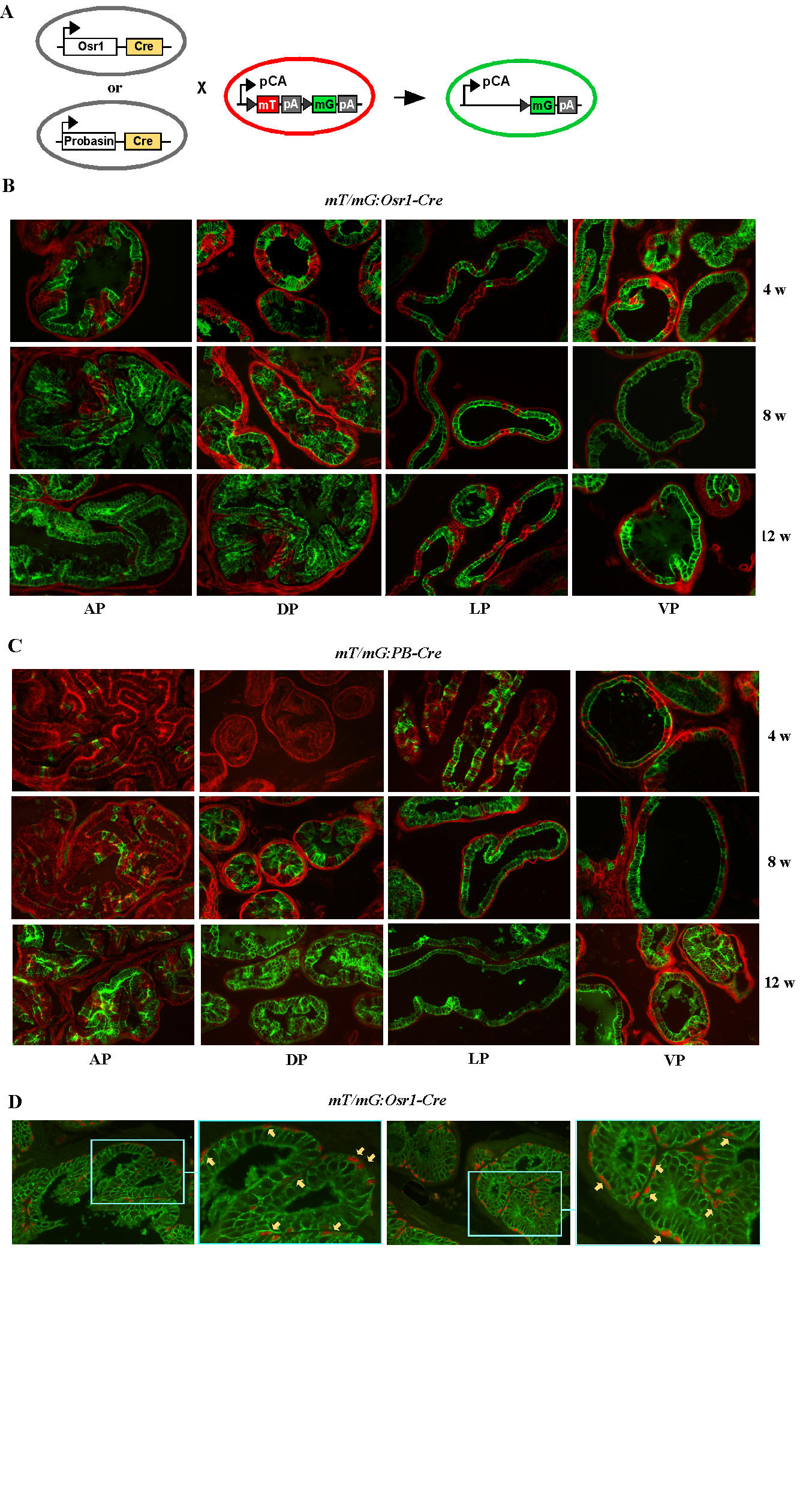

Supplement: Figure S2 — Generating mT/mG reporter mouse strains. A. Schematic diagram of the mT/mG reporter construct before and after Cre-mediated recombination. B. Live whole mount and fixed prostate tissues isolated from 8–12 week old male mT/mG;Osr1-Cre mice were analyzed. Representative Images were taken from prostate lobes, anterior (AP), dorsal (DP), lateral (LP), and ventral prostate (VP) of a 8 week old mT/mG:Osr1-Cre mice. C. Images were taken from a 8-week old make mT/mG:PB-Cre mice. D. Prostate tissues samples isolated from mT/mG:Osr1-Cre mice (B) were stained with the p63 antibody (red). Double p63 (red) and mG (green) positive cells are label with yellow arrows. (TIF) [file pone.0053476.s002.tif]
